# Strategy for Accurate Detection of *Escherichia coli* O157:H7 in Ground Pork Using a Lateral Flow Immunoassay

**DOI:** 10.3390/s17040753

**Published:** 2017-04-02

**Authors:** Song Cheng, Ming-hui Chen, Gang-gang Zhang, Zhi-biao Yu, Dao-feng Liu, Yong-hua Xiong, Hua Wei, Wei-hua Lai

**Affiliations:** 1 State Key Laboratory of Food Science and Technology, Nanchang University, Nanchang 330047, China; 15797769982@163.com (S.C.); minghuiai@sina.com (M.-h.C.); joezgg@sina.com (G.-g.Z.); defoelau@163.com (D.-f.L.); xiongyonghua@ncu.edu.cn (Y.-h.X.); weihua@ncu.edu.cn (H.W.); 2 Department of Science and Technology, Nanchang University, Nanchang 330047, China; yzb1156@126.com

**Keywords:** enrichment, *E. coli* O157:H7, ground pork sample, false positive

## Abstract

*Escherichia coli* O157:H7 is known to cause serious diseases including hemorrhagic colitis and hemolytic uremic syndrome. A gold nanoparticle lateral flow immunoassay (Au-LFIA) was used to detect *Escherichia coli* O157:H7 in ground pork samples. False-positive results were detected using Au-LFIA; a *Citrobacter freundii* strain was isolated from the ground pork samples and identified by using CHROmagar^TM^ plates, API 20E, and 16S RNA sequencing. Since *C. freundii* showed cross-reactivity with *E. coli* O157:H7 when Au-LFIA test strips were used, a novel method combining modified enrichment with a lateral flow immunoassay for accurate and convenient detection of *E. coli* O157:H7 in ground pork was developed in this study to minimize these false positives. MacConkey broth was optimized for *E. coli* O157:H7 enrichment and *C. freundii* inhibition by the addition of 5 mg/L potassium tellurite and 0.10 mg/L cefixime. Using the proposed modified enrichment procedure, the false-positive rate of ground pork samples spiked with 100 CFU/g *C. freundii* decreased to 5%.

## 1. Introduction

*Escherichia coli* O157:H7 is a dangerous foodborne pathogen because of its low infectious dose (minimum 10 cells) and high pathogenicity (diarrhea, hemorrhagic colitis, and hemolytic uremic syndrome) [1]. The “gold standard” for detecting *E. coli* O157:H7 in food samples is a traditional separation-identification method which is time-consuming (at least five days) and laborious (involves pre-enrichment, selective enrichment, culture isolation, and identification). Other methods, including the polymerase chain reaction (PCR) [2,3] and enzyme-linked immunosorbent assay (ELISA) [4,5], also may require laborious procedures and expensive instruments.

Lateral flow immunoassay (LFIA), which is widely used in the field of food safety, presents a number of advantages, including ease of use, rapidity, and sensitivity [6]. However, LFIA-based methods have the disadvantage that the antibody used in the immunoassay can show cross-reactivity with bacteria other than the target [7,8].

**Figure 1:**
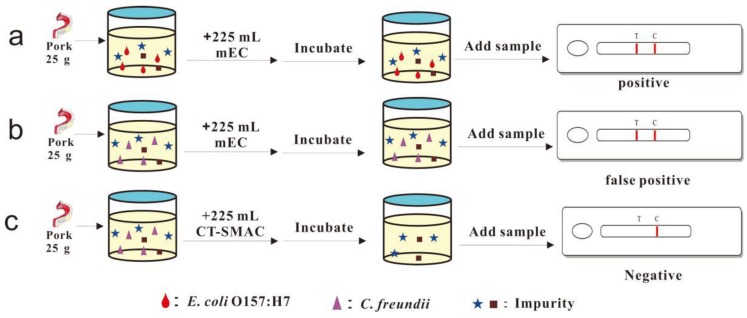
Overall process of the immunochromatographic assay for detecting *E. coli* O157:H7.

A gold nanoparticle LFIA (Au-LFIA) method was previously optimized for detecting *Escherichia coli* O157:H7 with high sensitivity [9,10,11,12,13,14,15]. Detection of *E. coli* O157:H7 in uninoculated ground pork samples using Au-LFIA test strips prepared in our laboratory in combination with the enrichment in modified *E.Coli* (EC) broth yielded positive results. In this study, the reasons behind the false-positive results obtained when Au-LFIA test strips are used to detect *E. coli* O157:H7 in ground pork were determined. A novel method combining modified enrichment with Au-LFIA for accurate detection of *E. coli* O157:H7 in ground pork was developed to minimize false positives (Figure 1).

## 2. Materials and Methods

### 2.1. Materials

Bacterial strains: The five *E. coli* O157:H7 strains and 40 non–*E. coli* O157:H7 strains used in this study are described in Table 1. Twenty ground pork samples were purchased from Guohong Pork Slaughterhouse (Nanchang, China), and another 20 ground pork samples were purchased from local supermarkets in Nanchang, China. A homogenizer and stomacher bags were purchased from Voshin Instruments Co., Ltd. (Wuxi, China). Nitrocellulose membrane, sample pad, conjugate release pad, and absorbent pad were obtained from Millipore (Bedford, MA, USA). Anti-*E. coli* O157:H7 monoclonal antibody (mAb, 10C5-H3-B6), anti-*E. coli* O157:H7 polyclonal antibodies (pAb), and goat anti-mouse antibody pAb were obtained from Wuxi Zodoboer Biotech. Co., Ltd. (Wuxi, China). Novobiocin, modified EC (mEC) culture medium, potassium tellurite, cefixime, and MacConkey Broth (CT-SMAC) culture medium were purchased from Beijing Land Bridge Technology Co., Ltd. (Beijing, China). Colloidal gold test strip reader was obtained from Suzhou Helmen Precision Instrument Co., Ltd. (Suzhou, China). CHROmagar^TM^ O157 agar for *E. coli* O157 was purchased from CHROMagar (Paris, France). API 20E test strips were purchased from Biomerieux (Lyon, France). Then 16S RNA sequencing was performed by Genscipt Company (Nanjing, China).

### 2.2. Preparation of Au-LFIA Test Strips

Au-LFIA test strips to detect *E. coli* O157:H7 were prepared in laboratory as described previously [14]. The test strip consisted of sample pad, conjugate pad, nitrocellulose membrane (NC), and absorbent pad. Anti-*E. coli* O157:H7 polyclonal antibody was applied to the nitrocellulose membrane as the test line. Goat anti-mouse antibody was applied to the nitrocellulose membrane as the control lines. Anti-*E. coli* O157:H7 monoclonal antibody-AuNPs complex was applied to a conjugate pad.

### 2.3. Pretreatment of Ground Pork Samples

Twenty ground pork samples were tested negative for *E. coli* O157:H7. Then 25 g of these ground pork samples was transferred into a stomacher bag as negative sample. Another 25 g of these ground pork samples was transferred into a stomacher bag and inoculated 2 CFU/g *E. coli* O157:H7 as positive samples.

### 2.4. Enrichment with Modified EC Broth and Evaluation by Using Au-LFIA Test Strip

Twenty negative samples and 20 positive samples (25 g) were mixed with 225 mL of modified EC broth containing 20 mg/L of novobiocin respectively, and stomached (Seward 400 Stomacher, Norfolk, UK) for 2 min. All of the samples were incubated at 37 °C with shaking at 160 r/min for 12 h. One hundred microliters of the 40 enriched broths of the ground pork samples was respectively added to the sample pad of Au-LFIA test strips for detection. When the target analyte (*E. coli* O157:H7) was added onto the sample pad of the test strip, it flowed laterally through the test strip. The *E. coli* O157:H7 interacted with the antibody-AuNPs complex in the conjugate pad, and aggregated subsequently at the T line because of the specific interaction between the *E. coli* O157:H7 and the antibody, which leads to a red color of the T line. Two visual bands (control line and test line) indicated a positive result. One visual band (control line) indicated a negative test result. The colored gold-antibody conjugate should bind to the control line and form a red-colored band regardless of the presence of *E. coli* O157:H7. The signal intensity of the test line can be detected by colloidal gold test strip reader. When the sample is positive, the signal intensity of the test line is equal or greater than 30.

### 2.5. Specificity of the Au-LFIA Test Strip

Five *E. coli* O157:H7 strains and 40 non- *E. coli* O157:H7 strains (Table 1) were cultured in Luria-Bertani medium (LB, Oxoid, Basingstoke, UK) at 37 °C for 20 h before use. Then 10^5^ CFU/mL of strains were prepared by serial dilutions of cultures in phosphate buffered saline (PBS, Sigma Chemical Company, St. Louis, MO, USA, 0.01 M, pH 7.4). One hundred microliters of the 45 strains were tested by the Au-LFIA test strip for evaluating the specificity of the method. The non–*E. coli* O157:H7 strains cannot interact with the antibody-labeled AuNPs and no red line develops at the test line.

### 2.6. Identification of the False-Positive Bacterium

CHROmagar^TM^ O157 agar without potassium tellurite and cefixime was used after ground pork sample enrichment. The enrichment broth was diluted 1:100,000 with sterile phosphate buffer, and the diluted broth (100 μL) was streaked onto CHROmagar^TM^ O157 agar. After incubation at 37 °C for 24 h, bacteria with different colors and colony morphology were inoculated into LB medium for enrichment and identified through API 20E and 16S RNA sequencing.

### 2.7. Cross Activity of *C. freundii* with Au-LFIA Strip

The isolated *C. freundii* strain was identified, inoculated into 250 mL of modified EC broth (10 CFU/mL), and incubated at 37 °C with shaking at 160 r/min for 12 h. The sample of the enrichment broth (100 μL) was added to the sample pad of Au-LFIA test strips. Three experiments were repeated.

### 2.8. Optimization of Enrichment Conditions

CT-SMAC culture medium with potassium tellurite and cefixime was optimized by adjusting the concentrations of potassium tellurite (2.50 mg/L, 3.75 mg/L, and 5.00 mg/L) and cefixime (0.05 mg/L, 0.075 mg/L, and 0.10 mg/L). Two portions of the ground pork samples that had been tested to be negative for *E. coli* O157:H7 and *C. freundii* were spiked with *E. coli* O157:H7 (2 CFU/g) and *C. freundii* which was identified in 2.6 (100 CFU/g). Two hundred and twenty-five milliliters of CT-SMAC with potassium tellurite and cefixime was mixed with 25 g of the ground pork samples and stomached for 2 min. All of the test samples were incubated at 37 °C with shaking at 160 r/min for 12 h.

### 2.9. Evaluation of the Optimized Enrichment by Using Au-LFIA Test Strip

Twenty negative ground pork samples were spiked with 100 CFU/g *C. freundii* as negative samples, while another 20 negative ground pork samples were spiked with 2 CFU/g *E. coli* O157:H7 as positive samples. Twenty-five grams of these 40 samples were respectively mixed with 225 mL of the optimized CT-SMAC culture medium and stomached for 2 min. All of the samples were incubated at 37 °C with shaking at 160 r/min for 12 h. One hundred microliters of the 40 enrichment broth of the ground pork samples were then respectively added to the sample pad of Au-LFIA test strips for detection.

## 3. Results and Discussion

### 3.1. Specificity of the Au-LFIA

The results showed that the five strains of *E. coli* O157:H7 were detected successfully, and no cross-reaction with the other 40 strains was observed (Table 1).

### 3.2. False-Positive Result of Au-LFIA Test Strip

Forty inoculated positive ground pork samples (20 from the slaughterhouse and 20 from the supermarket) and 40 negative control ground pork samples (20 from the slaughterhouse and 20 from the supermarket) were enriched by using modified EC broth. The samples were then tested for the presence of *E. coli* O157:H7 by using Au-LFIA test strips. Forty samples from the slaughterhouse, including 20 positive samples and 20 negative samples, yielded positive results. Twenty positive samples from supermarket showed positive test results. As well, eight out of the 20 negative samples from the supermarket showed positive test results, whereas 12 out of 20 uninoculated samples showed negative test results (Table 2).

The results obtained from Au-LFIA test strips indicated that some bacterial species in the ground pork samples exhibit cross-reactivity with *E. coli* O157:H7 (Figure S1). All 20 negative samples from the slaughterhouse showed false positives, whereas only eight of the 20 negative samples from the supermarket revealed false positives.

### 3.3. Isolation and Identification of *C. freundii* from Incubated Broth

The diluted modified EC broth (100 µL) was streaked onto CHROmagar^TM^ O157 agar. After incubation at 37 °C for 24 h, colonies with different colors and colony morphologies were produced. *E. coli* O157:H7 appeared mauve in CHROmagar^TM^ plates (Figure S2A), while some non-O157 bacteria showed other colors. The bacteria in 14 negative samples with false-positive Au-LFIA results were isolated using the CHROmagar^TM^ plate. These bacteria exhibited cross-reactivity with *E. coli* O157:H7 when the Au-LFIA test strips were used for detection and appeared blue on the CHROmagar^TM^ plate (Figure S2B). The bacteria producing false positives were identified by API 20E (Figure S3), and one bacterium, namely *C. freundii*, was identified through 16S RNA sequencing (Supplementary Material 4). The nucleotide sequence of *C. freundii* was identified with 99.9% accuracy by NCBI Blast, and the results obtained agreed with the API 20E findings.

### 3.4. Cross-Reactivity of *C. freundii* with Au-LFIA Strip

The positive results indicated that the Au-LFIA test strips for detecting *E. coli* O157:H7 exhibited cross-reactivity with *C. freundii.*

### 3.5. Optimization of the Modified Culture Medium for *C. freundii* Inhibition

**Figure 2:**
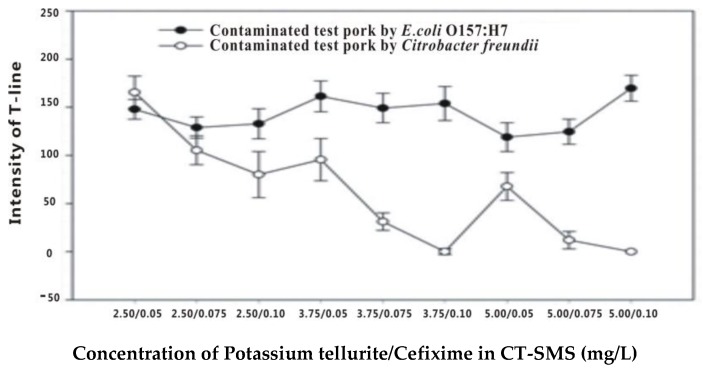
Signal intensities of the Au-LFIA strip test lines with pork broth samples spiked with *E. coli* O157:H7 and *C. freundii* (n = 3).

A series of various potassium tellurite and cefixime concentrations in CT-SMAC were studied to demonstrate the inhibition of *C. freundii* (Figure 2). When the potassium tellurite and cefixime concentrations in CT-SMAC were 5 and 0.10 mg/L, respectively, *C. freundii* did not multiply and *E. coli* O157:H7 was enriched. One hundred microliters of the enrichment broth of the ground pork samples was added to the sample pad of Au-LFIA test strips prepared in our laboratory. The signal intensities of the test lines of the strips with ground pork broth samples spiked with *E. coli* O157:H7 and *C. freundii* were 175 and 0, respectively.

### 3.6. Evaluation of Modified CT-SMAC in Ground Pork Test

Twenty ground pork samples that were negative for *E. coli* O157:H7 and *C. freundii* were spiked with 100 CFU/g *C. freundii* as negative controls and 2 CFU/g *E. coli* O157:H7 as positive controls. After enrichment by the developed culture medium, 100 µL of the enrichment broth of the ground pork samples was detected using Au-LFIA test strips, as well as CHROmagar^TM^ plates. Results (Table 3) indicated that the modified CT-SMAC was suitable for *E. coli* O157:H7 enrichment in ground pork samples. Using this enrichment procedure, ground pork samples spiked with 2 CFU/g *E. coli* O157:H7 showed 100% positive results and only a 5% false-positive result from *C. freundii*.

Previous studies had shown that some strains of *C. freundii* presented cross-reactivity with anti-O157 sera [16,17]. With the developed enrichment procedure, Au-LFIA had good specificity. Bennett and Zadik have also obtained a good specificity result with potassium tellurite and cefixime [18,19]. Some scholars also acquired sensitive and specific results based on lectin recognition of *E. coli* O157:H7 [20].

## 4. Conclusions

In this study, *E. coli* O157:H7 in ground pork samples was detected by using Au-LFIA test strips. A large number of false-positive results were obtained. The *C. freundii* strain was isolated and identified from the ground pork samples and determined to induce these false positives. A modified enrichment procedure by the addition of 5 mg/L potassium tellurite and 0.10 mg/L cefixime was evaluated for the enrichment of *E. coli* O157:H7 and the inhibition of *C. freundii.* Combining the modified enrichment procedure with Au-LFIA, ground pork samples spiked with 2 CFU/g *E. coli* O157:H7 showed 100% positive results and a 5% false-positive result from *C. freundii*.

## Figures and Tables

**Table 1:** Specificity of the Au-LFIA strip to *E. coli* O157:H7.

Species	Source	Result (Approx. 10^5^ CFU/mL)
*E. coli* O157:H7	ATCC 43888	+
*E. coli* O157:H7	CMCC 44828	+
*E. coli* O157:H7	NCTC 12900	+
*E. coli* O157:H7	XY0540 ^a^	+
*E. coli* O157:H7	XY0480 ^a^	+
*Bacillus cereus*	CMCC 63303	−
*Bacillus cereus*	CMCC 63305	−
*Bacillus cereus*	SLK ^a^	−
*Bacillus licheniformis*	CMCC 63519	−
*Bacillus subtilis*	BD366 ^a^	−
*Candida albicans*	ATCC 10231	−
*Candida albicans*	Z1 ^a^	−
*Cronobacter cloacae*	CMCC 45301	−
*Cronobacter sakazakii*	CMCC 45401	−
*Cronobacter sakazakii*	CMCC 45402	−
enteropathogenic *E. coli*	CMCC 44496	−
*E. coli*	CMCC 44102	−
*E. coli*	ATCC 25922	−
*Lactobacillus bulgaricus*	F1 ^a^	−
*Listeria grayi*	ATCC 25401	−
*Listeria innocua*	ATCC 33090	−
*Listeria innocua*	ATCC 11288	−
*Listeria ivanovii*	ATCC 19119	−
*Listeria monocytogenes*	ATCC 13932	−
*Listeria monocytogenes*	CMCC 54001	−
*Listeria monocytogenes*	CMCC 54007	−
*Listeria welshimeri serovar* 6b	ATCC 35897	−
*Listeria seeligeri*	ATCC 35967	−
*Micrococcus luteus*	CMCC 28001	−
*Proteus vulgaris*	CMCC 49027	−
*Pseudomonas aeruginosa*	ATCC 27853	−
*Pseudomonas aeruginosa*	CMCC 10104	−
*Salmonella anatum*	ATCC 9270	−
*Salmonella choleraesuis*	ATCC 13312	−
*Salmonella choleraesuis*	CICC 21493	−
*Salmonella enteritidis*	ATCC 13076	−
*Salmonella paratyphi* A	ATCC 9150	−
*Salmonella enterica*	ATCC 10708	−
*Salmonella typhimurium*	ATCC 13311	−
*Serratia marcescens*	CMCC 41002	−
*Shigella flexneri*	ATCC 12022	−
*Shigella sonnei*	CMCC 51592	−
*Staphylococcus aureus*	CMCC 26001	−
*Staphylococcus aureus*	CMCC 26003	−
*Vibrio parahaemolyticus*	CGMCC 1.1997	−

^a^ Strains were from Jiangxi Province Center for Disease Control and Prevention; “+”: positive result, “−”: negative result.

**Table 2:** Results of ground pork samples detected using Au-LFIA test strips.

Samples		Negative Result of Au-LFIA	Positive Result of Au-LFIA
Samples from the slaughterhouse	10 positive samples	0	20
	10 negative samples	0	20
Samples from the supermarket	10 positive samples	0	20
	10 negative samples	12	8

Positive samples are inoculated with 2 CFU/g *E. coli* O157:H7; negative samples are without inoculated with *E. coli* O157:H7.

**Table 3:** Evaluation of modified CT-SMAC in ground pork samples.

Pork Samples	CHROmagar^TM^ Plates		Au-LFIA Test Strip	
	+	−	+	−
Positive samples (*E. coli* O157:H7) (*n* = 20)	20	0	20	0
Negative samples (*C. freundii*) (*n* = 20)	0	20	1	19

“+”: positive result, “−”: negative result.

## Supplementary Materials

The following are available online at http://www.mdpi.com/1424-8220/17/4/753/s1, Figure S1: Detection results of the Au-LFIA test strips, Figure S2: CHROmagarTM plates used to isolate bacteria from the enrichment broth. (A) E. coli O157:H7. (B) Cross-reactive bacterium, Figure S3: Identification results of the cross-reactive bacterium by API 20E, Supplementary materials 4: Nucleotide sequence of 16S RNA of Citrobacter freundii.
