# Supplementary material for: Strategy for Accurate Detection of Escherichia coli O157:H7 in Ground Pork Using a Lateral Flow Immunoassay
Source: Sensors (Basel). 2017 Apr 2;17(4):753. doi: 10.3390/s17040753 (PMC5421713; doi:10.3390/s17040753)
Supplement: Supplementary file 1 [file sensors-17-00753-s001.pdf]

Supplementary Materials

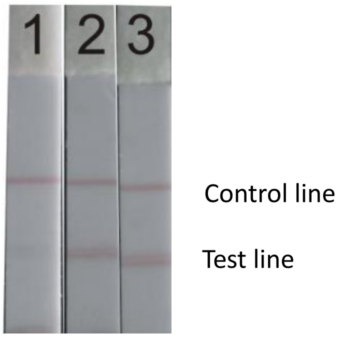

Figure S1. Detection results of the Au-LFIA test strips.

Strip Nos. 1 (mEC medium), 2 (false positive), and 3 (positive)

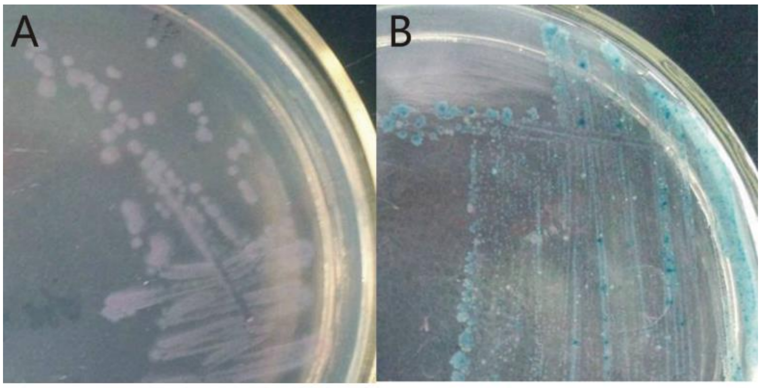

Figure S2. CHROmagarTM plates used to isolate bacteria from the enrichment broth. (A) *E. coli* O157:H7. (B) Cross-reactive bacterium.

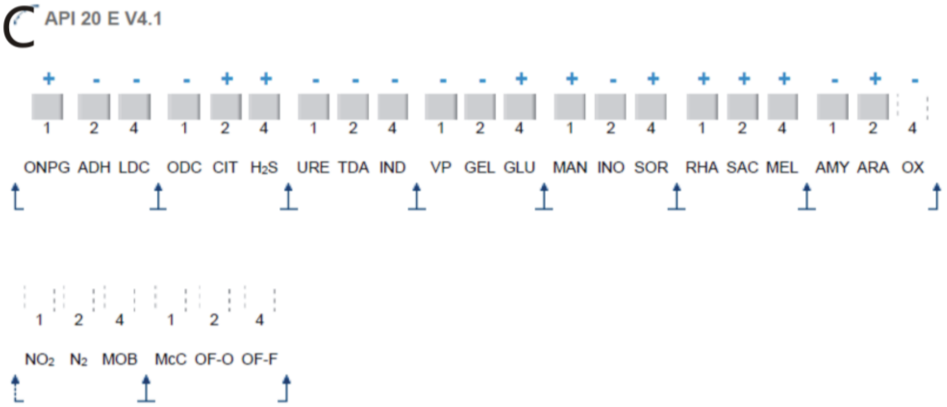

Figure S3. Identification results of the cross-reactive bacterium by API 20E.

Supplementary material 4
Nucleotide sequence of 16S RNA of *Citrobacter freundii*

TGCAAGTCGAACGGTAGCACAGAGGAGCTTGCTCCTTGGGTGACGAGTGGCGGACGG
GTGAGTAATGTCTGGGAAACTGCCCGATGGAGGGGGATAACTACTGGAAACGGTAGCT
AATACCGCATAACGTCGCAAGACCAAAGAGGGGGACCTTCGGGCCTCTTGCCATCGGA
TGTGCCCAGATGGGATTAGCTAGTAGGTGGGGTAACGGCTCACCTAGGCGACGATCCC
TAGCTGGTCTGAGAGGATGACCAGCCACACTGGAACTGAGACACGGTCCAGACTCCT
ACGGGAGGCAGCAGTGGGGAATATTGCACAATGGGCGCAAGCCTGATGCAGCCATGC
CGCGTGTATGAAGAAGGCCTTCGGGTTGTAAAGTACTTTCAGCGAGGAGGAAGGTGTT
GTGGTTAATAACCGCAGCAATTGACGTTACTCGCAGAAGAAGCACCGGCTAACTCCGT
GCCAGCAGCCGCGGTAATACGGAGGGTGCAAGCGTTAATCGGAATTACTGGGCGTAAA
GCGCACGCAGGCGGTCTGTCAAGTCGGATGTGAAATCCCCGGGCTCAACCTGGGAAC
TGCATCCGAAACTGGCAGGCTAGAGTCTTGTAGAGGGGGGTAGAATTCCAGGTGTAGC
GGTGAAATGCGTAGAGATCTGGAGGAATACCGGTGGCGAAGGCGGCCCCCTGGACAA
AGACTGACGCTCAGGTGCGAAAGCGTGGGGAGCAAACAGGATTAGATACCCTGGTAG
TCCACGCCGTAAACGATGTCGACTTGGAGGTTGTGCCCTTGAGGCGTGGCTTCCGGAG
CTAACGCGTTAAGTCGACCGCCTGGGGAGTACGGCCGCAAGGTTAAAACTCAAATGAA
TTGACGGGGGCCCGCACAAGCGGTGGAGCATGTGGTTTAATTCGATGCAACGCGAAG
AACCTTACCTACTCTTGACATCCAGAGAACTTAGCAGAGATGCTTTGGTGCCTTCGGGA
ACTCTGAGACAGGTGCTGCATGGCTGTCGTCAGCTCGTGTTGTGAAATGTTGGGTTAA
GTCCCGCAACGAGCGCAACCCTTATCCTTTGTTGCCAGCGGTTAGGCCGGGAACTCAA
AGGAGACTGCCAGTGATAAACTGGAGGAAGGTGGGGATGACGTCAAGTCATCATGGC
CCTTACGAGTAGGGCTACACACGTGCTACAATGGCATATACAAAGAGAAGCGACCTCG
CGAGAGCAAGCGGACCTCATAAAGTATGTCGTAGTCCGGATTGGAGTCTGCAACTCGA
CTCCATGAAGTCGGAATCGCTAGTAATCGTGGATCAGAATGCCACGGTGAATACGTTCC
CGGGCCTTGTACACACCGCCCGTCACACCATGGGAGTGGGTTGCAAAAGAAGTAGGT
AGCTTAACCTTCGGGAGGGC
